# Relation of Infant Diet to Gastro-Intestinal Diseases

**Published:** 1875-12

**Authors:** T. Curtis Smith

**Affiliations:** Middleport, Ohio


VOL. V.—DECEMBER, 1875.—NO. 12.

### Original Communications.

### RELATION OF INFANT DIET TO GASTRO-INTESTINAL DISEASES.

BY T. CURTIS SMITH, M. D., MIDDLEPORT, OHIO.

*Read before the Ohio Valley Medical Association, at Ironton, Ohio, Oct. 13, 1875.*

*Mr. President and Gentlemen:*

At the risk of being charged with performing a needless task, I have concluded to give you a brief paper on the subject. True, much has already been said and written, and a vast number of our medical brethren seem to thoroughly understand the subject, many no doubt far better than your essayist, but the vast importance of the subject is such, that I am led to believe that we cannot too often review it, and teach it "line upon line, and precept upon precept," not only to the profession proper, but to the laity also.

When we consider the lamentable fact, that even in our day of efforts at good hygiene in the way of cleanliness, ventilation &c., over thirty per cent. of the children born in this and other civilized countries die before they reach the age of five years, it becomes at once a question of vast importance to us as a profession, to the parents of the children, and to the State, as a matter bearing upon political economy. A question worthy of consideration as to the cause of this lamentable mortality, and the vast amount of suffering which these little innocents endure during their brief existence.

The statement that thirty per cent. die at so early an age has and can be plainly demonstrated from the statistics of this and other countries. For instance, Dr. E. Ballard, for the five years of 1863 to 1868, gave the following official table from the mortality reports of England, of children under five. In this report we find the following:*

| | |
|---|---|
| Deaths from causes not specified | 12,458 |
| Sudden deaths, causes not ascertained | 3,879 |
| Deaths from violence | 8,398 |
| Want of breast milk | 6,595 |
| Diarrhea | 54,274 |
| Thrash | 5,060 |
| Atrophy and debility | 107,825 |
| Teething | 10,459 |
| Convulsions | 105,249 |
| Total | 314,242 |

Now if we sum up the last six items, and all of these are quite generally connected, directly or indirectly, with some form of alimentary derangement, we have 289,507 deaths from these, leaving but 24,735 deaths from the first three items of cause. Now I think any one will certainly grant that a large per cent. of the deaths under the first two items were probably from diseases of the alimentary tract, thus increasing the per cent. of mortality of digestive derangements. But if we take the percentage of deaths caused by the last six items, we find it to be over 85 per cent. This corresponds very closely with the statements of Burns in 1823, and Prof. Thomas C. James, (Diseases of Women and Children), of Condee in 1843, Merriman, Combe, and a host of others, as to the mortality of infants.

But probably it would be unfair to attribute the large per cent. to faulty diet alone, as there are many cases of alimentary diseases caused by surrounding filth, bad ventillation, malaria, cold &c. But on the other hand there are very many instances inwhich children suffering from digestive disorders, are attacked with other diseases

As given by B. F. Dawson, *Amer. Jour. Obstet. and Dis. Chil.*, Aug., 1875, p. 218.

and die, and their deaths are attributed to the last disease affecting them, whereas, had the children been in good health and well nourished, they could have easily withstood the disease last affecting them with impunity. Again, the very fact that they are reduced in physical stamina, in consequence of their illy nourished condition, opens the way for the ready attack of many fatal maladies that children might otherwise escape. Thus an attack of pneumonia, bronchitis, or fever, cannot be as safely endured in a feebly nourished child as in one in the reverse condition. Such being the fact, many infants succumb indirectly to the influence of malnutrition, resulting from improper diet, in either quantity or quality. From these facts and the known direct deaths from intestinal disorders, caused by improper feeding, we may certainly and regretfully claim a mortality of fifty per cent. in infants and children (that die) as due to malnutrition, nearly all traceable to the single cause alluded to. According to the report of Board of Health, of Philadelphia, 36.83 per cent. of the children (J. S. Parry, Infant Mortality, 1871) die before reaching their fifth year. In New York, "more than one third of all the infants born * * * die under the age of five years;" "fifty-three per cent. of the total number of deaths under the age of five years, and 26 per cent. under the age of one year" (J Lewis Smith). "Of 9,873 children who died in Massachusetts in 1870 under five years of age, more than one-half of the deaths were due to affections of the digestive organs" (Chas. E. Buckingham, Proper Treatment of Children, 1873). Prof. B. F. Dawson claims that, "even if we take from above figures (of the table before given) those deaths due to disorders of the digestive organs and insufficient alimentation, and accrediting, as in my opinion, it is but right to do, only one-half of the deaths from thrash, atrophy, teething, and convulsions to the same class, we still have out of 314,242 deaths, more than one-half, or 175,208 of infants destroyed by gastro-intestinal disorders" (*Amer. Jour. Obs. and Dis. Chil.* in 1875, p. 219). Statistics from large cities and vast scopes of territory can here be multiplied, but this we deem a needless task. It is not fair to include all cases of intestinal diseases under the cause of improper feeding, for we are all well aware of the evil results upon the health of children of impure air, continued dampness, insufficient clothing, filth, cold, &c., but after making a large allowance for all of these, and for congenital feebleness, we still have numerous high authorities for the statement that one-half of the deaths of young children and infants are due to faulty alimentation.

There is no question but that the mothers milk is the proper diet for the infant, but cases are very numerous where this cannot be supplied at all, or if so, only in sufficient quantity, and more often defective in quality, than we are wont to believe. A very fruitful source of infantile intestinal disorders is the attention given them in the way of diet while awaiting the first appearance of the mothers milk immediately after confinement. Very often the nurse, or some well meaning, but officious old lady, persists that the child needs some "pap," an article rich in starch, and which the infant can little more digest than it could a block of wood, for the reason that the salivary glands not yet being developed, fail to secrete saliva, which is so necessery for the conversion of the starch into sugar. Often they will feed it lard or butter, and sugar, a most disgusting mixture, and one that would turn the stomach of most any adult. From such pernicious feeding many children die immediately or remotely. On the other extreme, a few insist that if the mother has no milk for the infant we must wait until it appears, and too often such waiting is prolonged until the infants have become so feeble they cannot drain the milk from the mipple and they soon die of inanition. A larger per cent. of infants die during the first month of existence than any other month of life, and the ratio decreases with the age for the first few months of life. How shall we remedy this? is a question worthy of thought. The mother's milk cannot be obtained. Starchy and very fatty foods are inadmissible on physiological grounds. Cow's milk, the most easily attainable aliment, is not always to be had, but, with a little diligent effort, can usually be reached. But it is too strong, it is thought. We must, therefore, make it as near the natural milk as possible, and if we do this we shall come as near attaining the desired end as it is in our power. Unfortunately too many physicians, who ought to know better, think if they add plenty of water to cow's milk and sweeten it a little, their duty is done. Fortunately most infants will endure this diet for one or two days, the time necessary for the mother's milk to appear. But if the infant must needs continue to be fed on such diet thus prepared, it will not fail in a very few days to show distinctly and in unmistakable symptomatic language that such diet is improper, and that it is starving, though the stomach is filled to repletion. What then shall we do? If we look the matter fairly and considerately in the face, in the light of attainable knowledge of physiology and chemistry, we may easily arrive at an approximately correct conclusion as to the proper course to pursue in these cases. The cow's milk must be used, as it is the only milk readily obtained in the most parts of this country; but it must needs undergo some preparation. If we refer to the relative chemistry of cow's milk and woman's milk, as given by Vernois and Beguerel, we find cow's milk nearly the same in specific quanity; that it contains to the 1000 parts 25.02 parts less of water, 25.02 parts more of solids, 4.39 less of sugar, 15.91 more of casien, 9.46 more butter, and nearly five times more salts than the woman's milk. Now can we not right this up so as to make it very nearly level in proportion to the mother's milk. If we add a trifle over three per cent. of water to cow's milk we have these proportions equal; then if we add a trifling amount of sugar (sugar of milk preferable), we have leveled this up.* But the caseine in the cow's milk is too much and we cannot extract it, and so also is the butter. How shall we manage this? A moment's thought tells us that the casein of the cow's milk coagulates into large, firm lumps, while that of a woman's milk coagulates in small, soft, flaky lumps; hence the former is much more difficult of digestion than the latter, and will therefore be much more apt to disagree with the infant's feeble stomach, and such is the fact. Out of an imperfect knowledge of these points of difference in the milks has grown the practice of adding one-third to four-fifths water to cow's milk. But a mere glance will show that this does not help us out of the milk trouble, for the water does not soften the curd of the milk one whit; and in order to have the child take sufficient food, it must needs

*The salts, especially the phosphates in the cow's milk, is about five times as great as in the woman's milk, and these cannot well be equalized, but fortunately their excess is not sufficient to materially interfere with the growth and development of all the tissues, unless possibly it interferes with the correct formation and development of cartilageous tissues. The damage to the system is at least far less than we would expect reasonably, from a great lack of these salts drink a proportionately large quantity of water. If the milk be diluted one-half, it must drink twice the quantity of fluid to get a sufficient amount of nutriment to sustain it properly. Not only this, but the curd is not softened by the water nor rendered more digestible, for the water is soon absorbed or passed out of the stomach, leaving the curd behind. It has been thought that the firm curd of the cow's milk is softened by adding starchy food to it, and this is effected by the particles of starch interposing themselves between the particles or layers forming the curd. Experience has not proved that starchy food has ever added anything to the digestibility of the curd of cow's milk in the young infant's stomach. But if we grant that it does render the curd more easy of digestion, how is the stomach to digest the foods. The infant does not secrete saliva for the first two months of its life, and saliva is very essential to the digestion of starchy food? After the child has become older than two months, the starchy food is still detrimental, from the fact that the system cannot appropriate it without suffering from the non-removal of other principles necessary to its health. "In nutrition it is important that tissue change should be rapid, and in young children, where development, as well as growth, is so brisk, this is of especial importance. It is effected by the oxidation of old material, which is then removed to be replaced by new matter. For rapid change, therefore, it is indispensible that no needless impediment should exist to the free oxidation of the tissues. Now starches and sugars into which the starches are converted by digestion, have a greater affinity for oxygen than albuminates; they, therefore, tend to appropriate the oxygen which is required for the removal of waste matters, and so prevent the proper changes from taking place. For this reason alone, and without any reference to their indigestible properties, they form a very unsuitable diet for a young child." (*Eustace Smith.*)

From this it is plainly seen that the almost universal habit of adding a little flour, stale bread, cracker, potato, &c., to the milk to be fed to a young infant is unphysiological, and therefore, as experience has proven, is more or less decidedly detrimental. True,

---

in the cow's milk, as such a lack would be liable to lead to an imperfect development of all the osseous and cartilagenous structures, and probably deficient generation of healthy nerve cells.

the child often lives under such a course of diet; rarely, it thrives; but it does so in spite of the starchy food, and by its own inherent power to overcome the obstacle thus placed in its way. Well, if water will not soften the curd, or coagulate casein of cows' milk, and its addition, to any considerable extent, is so decidedly detrimental, by its needlessly over-distending the stomach in order to get a sufficiency of food, and starch is not only totally indigestible to the very young infant, and more or less so to the older infants, and withal fails to soften the curd, besides proving detrimental by its stronger affinity for oxygen than for the albuminates, what will we use to accomplish the desired end? Here the difficulty looks us squarely in the face. The habit of feeding infants on milk and water slops has attained such a strong hold upon the profession, and, through them, upon the laity, that a physician is looked upon by many as a fool or an ignoramous if he departs from this old beaten path. Dr. Hiram Corson, of Conshohocton, Pennsylvania, in a valuable little reprint on this subject, sets forth some features of this evil in a strong light. He recommends simply "cow's milk itself." Now, this is decidedly good treatment in comparison with the plan of diluting the milk one-third, half or two-thirds, and, doubtless, this will very often lift a child from the state of enforced inanition which he so forcibly describes. But can we not do a little better than this? If we can find something that will soften the curd, or prevent it from forming in such hard lumps, we will have accomplished the end, for it is totally unnecessary to dilute cow's milk under the plea that "whole milk is too strong," or that it is "too rich;" such is not the fact. If, now, we take cow's milk, in its full richness, and add to each pint one to two ounces of cream, or in lieu of this a lump of fresh butter the size of a hazlenut, or an equal quantity of good oil—almond or pure olive oil are very good—the curd will be sufficiently softened, or prevented from forming such hard masses that it will be quite as readily digested as the mother's milk. But I hear almost invariably from mothers the expression, "that it is too rich—the child cannot bear it." Experience has proven to me that they do bear it, and thrive upon it, after fruitless attempts by the old plan have utterly failed. So that, instead of weakening the milk, I deem it best to add, not to exceed five per cent. of water, a trifle of sugar—sugar of milk is best—and to this add the cream, or some of the fatty elements above named. Only a short time since, I was called to see an infant two months old. It looked like a little dried up old man. At its birth it was as fat and lusty an infant as one would care to see. It nursed the mother a month, when the milk failed both in quantity and quality. This compelled artificial feeding, and the *inevitable* half-milk and half-water slop was resorted to, through the medium of the nursing bottle. The child had been fed on this one month, with the occasional addition of grated cracker to the mixture. When I saw it, the flesh had quite disappeared from its bones, its face was wrinkled and withered, belly bloated, skin on the extremities loose as a shirt, and the whole surface was of a dark, livid hue. The child fretted continually, wanted the bottle all the time—was constantly whining when its mouth was not filled. Every few minutes, or once an hour at least, the bowels were moved, the passages being slimy, with lumps of undigested curd mixed through them. Occasionally it would vomit curdled milk. Here was an infant actually starving with its stomach constantly filled to repletion—constantly! The mother had given it a full dose of oil, which had operated well, and no doubt had assisted, in its course through the stomach and bowels, in softening some of the curd and rendering it assimilable. I at once examined the milk, found it of good quality, and from a healthy cow. I took a pint of it, added to it five tablespoonfuls of rich cream, a half ounce of hot water, and a little sugar—about a teaspoonful; the water brought it up to the proper temperature. Seeing that the nursing-bottle was thoroughly cleansed, and giving directions how to keep it so, as well as to the necessity of frequently cleansing it, I had the child fed all it would take, the mother all the time protesting that the rich milk would kill it. When the child had taken near a gill, it dropped off in a quiet sleep, which was stated to be the first quiet sleep it had enjoyed for many days. The child was fed with this mixture every four hours regularly through the day, all it would take, and once or twice through the night. Not a particle of medicine in any form was given to it. The good effects of the change were apparent in a very few days, and the infant continued to thrive and do well on the mixture, taken in amount to the full of satisfaction.

Dr. Corson insists, as do other writers upon these points, viz., give enough, give it regularly, and do not dilute the milk.

He relates many cases similar to the one I have given above, and doubtless many of us can refer to many similar ones. I have, in other times, used and recommended the milk and water slop, but have long since discarded it as unphysiological, and as yielding unsatisfactory results.

Other forms of food have been recommended for young infants, such as Liebig's; but these are not half of the time attainable, or their preparation is so difficult as to render it a great obstacle to their use. Nothing will so well answer the place of the mother's milk—which, of course, is always to be preferred, if attainable—as that from a good healthy cow, so prepared as to have the curd rendered digestible by the small infant, or any infant under one year.

It is doubtless true that a proportion of infants will digest pure cow's milk, undiluted, after they have attained the age of a few months, if it is given to them systematically, *i. e.*, at regular and proper intervals, instead of being crammed into the overworked stomach whenever the child is peevish or whimpers a little. An adult could not endure such irregular feeding, with any kind of food, for a long continued period, much less an infant. But proportionately few children will endure the milk and water slop for any considerable period, without suffering from indigestion, colic, diarrhœa, emaciation, etc. In order to get from one to three pints of milk, it must needs drink twice that quantity of fluid; but finding this impossible, it must go on half fare, with a stomach weakened by an over-abundance of fluid. It is far better to give the milk in its natural concentrated form, than to over-crowd the stomach with water.

Dr. Corson justly remarks that not more than one mother in ten, and not many physicians, can state, with any degree of accuracy, how much milk an infant will drink, and must drink, in twenty-four hours, in order to thrive as it should. A very young infant will require a pint, and the quantity will be gradually increased, until they will take one and a half to two and a half quarts daily. As infants increase in age they may gradually be allowed other articles of food, but these should be mostly in the way of animal broths, in preference to amylaceous diet, which latter should only be allowed in very sparing quantities during the first year. Dr. Jacobi recommends that the milk be diluted with barley-water, giving as a reason that it is not only nourishing, but that the starch it contains is in such a very finely divided state that it not only tends to soften the curd by its easy interposition between its layers, but is also readily digested. This preparation seems to have succeeded well in his hands, and in that of others; but still the same objection to it attains that has been heretofore stated. Many children will, in the face of a want of heart-milk, get along, because the mother soon leaves it to suck a rind of meat, or something of this kind, by which it obtains considerable nutriment, and it soon learns to clamor for it whenever brought within view of the table. Others still will thrive in spite of the habit of feeding them on starchy foods at every meal, and in fact every other article of diet on the table; but where children are thus fed they are often ill, and the attendance of the physician is often called. In a vast majority of these cases infants need little or no medicine; simply correct the diet and it soon recovers. If children are fed, clothed, warmed, aired, etc., upon the principle of good hygiene and physiology, there is no reason to expect them to be ill any, or but little oftener than adults, and the great bugbear of teething will be gone through without any noticeable trouble; and instead of many of them growing up weak, puny men and women, they will grow up strong and healthful.

Thousands of infants and young children die annually in our land in consequence of faulty alimentation, and we are not wholly blameless. Their improper diet causes indigestion, vomiting, diarrhœa, which finally becomes chronic; emaciation and atrophy result from the slow process of starvation; they are peevish, actually cross; suffer from colic, intestinal pain and irritation; sleep but little, and that little is disturbed. As emaciation proceeds, the skin assumes first a clayish hue, then becomes livid; the child breaks out in sores, and must have its blood doctored; glandular swellings put in an appearance; abscesses are liable to form all this time; the child pines and frets because its aliment is insufficient in quantity, or improper in quality; finally, unless rescued by some accidental use of proper food, or that directed to be given by an intelligent physician, it droops and dies. The doctor too often exclaims, "All was done that could be done for it," and the laity take up the saying and transport it around among themselves. Children are not safe from the dangers of faulty diet, in the directions above pointed out, until they have passed their second year, many not until much older; and it is no uncommon occurrence to find children, approaching the close of their first year, or past it, who are actually starving for want of sufficient or proper nutriment, because the mother's milk, upon which they are still dependent, is insufficient to supply the demand of the system. These often fall sick as above described, and are dosed heavily, often to death, while the cause of their illness goes unnoticed, and therefore unremoved.

Medication in many of these cases is often worse than useless until the cause is removed, and in very many cases no medication whatever is beneficial, other than as a placebo. A simple bitter and aromatic tonic, to give tone to the stomach, may sometimes be of advantage. Of these, probably salicine, combined with an aromatic, is among the best. One of the first items worthy of note is generally the vomiting of milk, or its passage from the bowels undigested. Small doses of oil are best to remove these, if needed; then the feeding should commence very gradually, by teaspoonfuls at first, or, if need be, entire rest may be given to the stomach for some hours.

On the point of rest for the stomach in these cases, a high authority* makes the following remarks, not altogether inapplicable here:

"In the foregoing remarks I do not wish to be understood as denying that very many cases of gastro-intestinal disorders are unavoidable, even with the utmost care, especially as premonitory or concomitant signs of other diseases; but I do wish to be understood as of the opinion, the result of experience, that the majority of infants and young children suffer and die from gastro-intestinal disorder because of the prevailing ignorance as to the requirements of the infant as to the quantity and quality of the nourishment requisite to maintain perfect health; and that, when such diseases do occur, it would save many an infant suffering and death if the stomach and bowels were given that rest for which they vainly ap-

* B. F. Dawson, of New York City.

peal, in a language too often unheeded—vomiting and diarrhœa."

In conclusion, we claim, 1st. That where infants must be artificially fed, that cow's milk, with the addition of three per cent. of water,† a little cream or pure oil, is best.

2. That starchy foods in any form are decidedly detrimental until the salivary glands are well developed, and also during rapid growth of the early months, for reasons before stated.

3. That young infants suffering from indigestion should have a period of rest for the stomach, and that on resuming its food it should receive it at regular intervals, and

4. When it is fed, it should receive enough to satisfy its hunger.

5. That children properly fed and clothed will be quite as apt to thrive, and grow up strong and healthy, as those nursed at the healthy breast.

6. The suffering and illness of infants at the period of dentition are due very largely to faulty diet.

7. That due attention to purity, quality and quantity of food, and to its being given at proper intervals, will be a great improvement on the plan of large and frequent medicinal dosing.

